# Laparoscopic wedge resection of a descending duodenal gastrointestinal stromal tumor under endoscopic nasobiliary drainage guidance: A case report

**DOI:** 10.1016/j.ijscr.2025.110877

**Published:** 2025-01-13

**Authors:** Woo Yong Lee

**Affiliations:** Department of Surgery, Inje University Haeundae Paik Hospital, 875 Haeundae-ro, Haeundae-gu, Busan 48108, Republic of Korea

**Keywords:** Gastrointestinal stromal tumors, Descending duodenum, Endoscopic nasobiliary drainage, Limited and minimally invasive surgery, Case report

## Abstract

**Introduction:**

Gastrointestinal stromal tumors (GIST), which occur anywhere in the gastrointestinal (GI) tract, typically occur in the stomach and small intestine but rarely in the duodenum. We present a case report wherein a descending duodenal GIST was treated with a limited, minimally invasive surgery after endoscopic nasobiliary drainage (ENBD) insertion.

**Presentation of case:**

A 67-year-old woman visited our hospital with an incidentally discovered duodenal tumor. Gastroduodenoscopy revealed a duodenal subepithelial tumor (SET) measuring approximately 2 cm in descending duodenum. Endoscopic ultrasound revealed a well-circumscribed, inhomogeneous hypoechoic lesion measuring approximately 17 × 4.6 mm, thought to arise from the muscularis layer. Computed tomography (CT) revealed an inhomogeneous enhancing mass with central necrosis, measuring approximately 2.7 cm, in the descending duodenum. Pathological findings from the bite-on-bite biopsy showed c-kit and DOG-1 positivity and CD34 and desmin negativity, leading to a GIST diagnosis. Laparoscopic wedge resection with preoperative ENBD insertion was planned due to the risk of pancreaticobiliary duct (PBD) damage during surgery because the lesion was located near the ampulla of Vater (AoV) and minor papilla. Surgery was performed using laparoscopic wedge resection without PBD injury. The patient was discharged 10 days post-surgery without complications.

**Discussion:**

Descending duodenal GIST is difficult to operate on with minimally invasive surgery. However, if the size is not excessive and the PBD is not involved, minimal and limited surgery is possible after ENBD insertion.

**Conclusion:**

We report the first case of limited and minimally invasive surgery followed by ENBD insertion in a rare descending duodenal GIST.

## Highlights

• A rare case of a descending duodenal GIST treated with minimally invasive surgery.
• Endoscopic nasobiliary drainage was inserted preoperatively to prevent damage.
• Laparoscopic wedge resection was successfully performed without complications.
• Maintaining negative margins is crucial for preserving organ function.
• This approach may facilitate limited surgeries for duodenal GISTs.

## 1 Introduction

Gastrointestinal stromal tumors (GIST) are treated by removing tumors without margin involvement [1]. For tumors amenable to limited resection, maintaining negative margins is the best treatment to maintain organ function. GISTs primarily occur in the stomach and small intestine, including the jejunum and ileum, but rarely in the duodenum [2].

Duodenal GISTs can occur in any section of the duodenum. Owing to the anatomical location of the duodenum, it is difficult to determine the method and extent of surgery, which is dependent on the tumor's size and location [3,4]. If the GIST occurs in descending duodenum, pancreaticoduodenectomy(PD) may be necessary. Even in cases wherein minimal resection is possible, pancreaticobiliary duct damage remains a risk.

In this case, we report a safe laparoscopic limited surgery of a descending duodenal GIST by preoperative endoscopic nasobiliary drain (ENBD) insertion to protect the pancreaticobiliary duct (PBD), which can be damaged during laparoscopy. This article has been reported in line with the SCARE criteria [5].

## 2 Case presentation

A 67-year-old woman visited our hospital with an incidentally discovered duodenal tumor. No unusual findings were observed in previous checkups, and the patient reported no gastrointestinal symptoms.

**Fig. 1:**
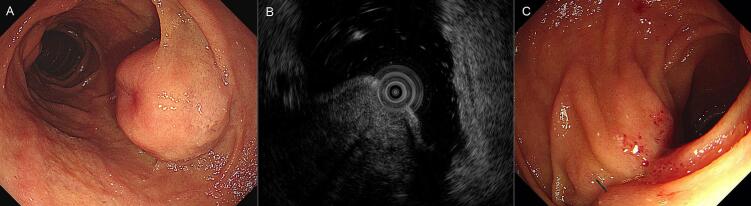
Endoscopy showing about 2-cm lobulated lesion with central depression at the second portion of duodenum (A). Endoscopic ultrasound shows an approximately 17 × 4.6 mm sized inhomogeneous hypoechoic lesion with a well-circumscribed margin that was thought to arise from the fourth layer (muscularis layer) (B). Three months later, there were no abnormalities or recurrences in the postoperative follow-up endoscopy (C).

**Fig. 2:**
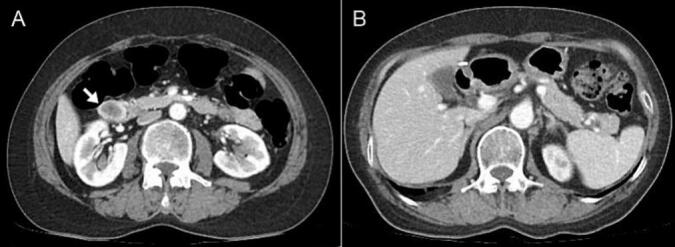
Abdominal computed tomography revealing a 2.7 cm sized inhomogeneous enhancing mass with central necrosis in duodenal 2nd portion without enlargement of the surrounding lymph nodes (A). Six months later, there were no abnormalities or recurrences in the postoperative follow-up computed tomography (B).

Gastroduodenoscopy revealed a duodenal subepithelial tumor (SET) measuring approximately 2 cm in the descending duodenum near the ampulla of Vater (AoV) (Fig. 1A). Endoscopic ultrasound (EUS) and computed tomography (CT) were performed to determine SET characteristics. EUS revealed an inhomogeneous hypoechoic lesion with a well-circumscribed margin measuring approximately 17 × 4.6 mm, thought to arise from the muscularis layer (Fig. 1B). A GIST or neuroendocrine tumor (NET) was suspected based on EUS findings. CT revealed an inhomogeneous enhancing mass with central necrosis (measuring approximately 2.7 cm) in the descending duodenum, suggesting that the GIST or NET was a subepithelial tumor without enlargement of the surrounding lymph nodes (Fig. 2A). A bite-on-bite biopsy was performed for histological confirmation, and the pathological results showed c-kit and DOG-1 positivity and CD34 and desmin negativity, resulting in a duodenal GIST diagnosis. Based on the above examination, it was thought to be a GIST with malignant potential, so surgical resection was decided.

Laparoscopic wedge resection was planned because of the limited resection. However, there was a potential risk of PBD damage during surgery because the tumor was located near the AOV and minor papilla. Therefore, we decided to insert the ENDB before surgery to prevent damage using a catheter as a guide. The procedure was performed using a standard duodenoscope (TJF-260 V; Olympus, Tokyo, Japan) under conscious sedation with midazolam and pethidine. Endoscopic sphincterotomy less than 3 mm was done with a sphincterotome (CleverCut 3 V; Olympus, Japan) after selective cannulation of the common bile duct (CBD), and visualization of CBD on fluoroscopy was undergone. After confirming no abnormal findings including choledocholithiasis and bile duct stricture on cholangiography, 5-Fr endoscopic naso-biliary drainage (ENBD) tube (Cook Medical, Bloomington, IN, USA) was placed in the intrahepatic duct. There were no special difficulties or complications such as bleeding or perforation during the procedure. To prevent pancreatitis after ERCP, intensive intravenous hydration with lactated Ringer's solution was provided. A series of laboratory tests were performed after the procedure, and it was confirmed that there was no evidence of side effects after ERCP, and the surgery was performed.

**Fig. 3:**
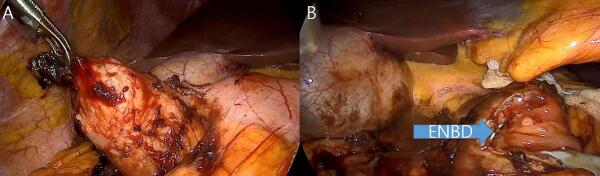
Surgical findings revealed an exoendophytic mass in the descending duodenum(A), laparoscopic wedge resection was performed on the duodenal wall, including the tumor during resection, the ENBD catheter was checked(B).

Surgical findings revealed an exoendophytic mass in the descending duodenum (Fig. 3A). Using LigaSure™, mobilization (Kocher maneuver) was performed on the descending and transverse duodenum, and wedge resection was performed on the duodenal wall, including the tumor. During resection, the ENBD catheter was checked to determine whether the PBD was damaged and the resection site was interrupted suture (Fig. 3B).

**Fig. 4:**
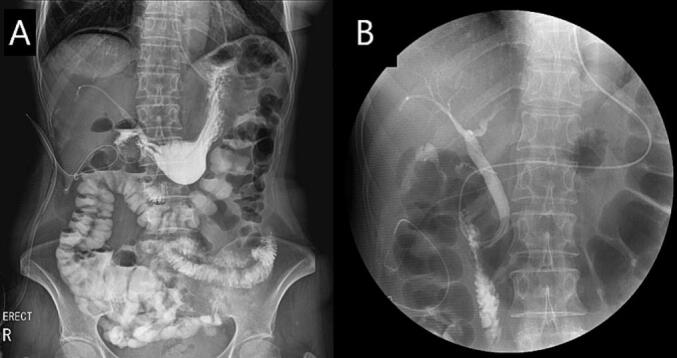
UGI series was showed to confirm that there was no anastomotic site leak (A), and an ENBD tubography was performed 2 days later to confirm that the PBD tract was undamaged (B).

Pathological examination revealed a tumor measuring 2.5 × 2 cm, mitotic count of 2/5 mm2(50HPF), c-kit positivity, and a clear surgical margin. According to the modified National Institutes of Health risk classification, the final diagnosis was GIST with a low risk of aggressive behavior [6]. On the 6th day post-surgery, a UGI series was performed to confirm that there was no anastomotic site leak (Fig. 4A), and an ENBD tubogram was performed 2 days later to confirm that the PBD tract was undamaged (Fig. 4B). ENBD was removed post-surgery because the tubography showed no specific findings in the CBD. The patient was discharged from the hospital 10 days post-operation as the patient exhibited no issues when consuming a general diet.

After 3 months, no abnormalities or recurrences were observed during postoperative follow-up examinations, including gastroduodenoscopy and CT (Figs. 1C, 2B). Gastroduodenoscopy revealed a wedge resection scar on the proximal side of the descending duodenum with no signs of recurrence. Afterwards, the patient was followed up with CT at 3- month intervals, and no recurrence was found.

## 3 Discussion

Gastrointestinal stromal tumors (GISTs) can occur anywhere in the gastrointestinal(GI) tract. However, it rarely occurs in the duodenum. Treatment of GIST is based on the complete removal of the tumor without margin involvement. Accurate preoperative diagnosis and staging of duodenal GIST are important to determine the appropriate treatment method [7].

Although gastroduodenoscopy is commonly used to evaluate the features of duodenal SETs and assess mucosal ulceration or bleeding, routine endoscopic forceps biopsy for histologic diagnosis is unreliable because of the submucosal location of GISTs and the risk of bleeding. EUS can also evaluate the internal echo pattern, exact size, shape, primary site, and vascularity of SETs. EUS-guided fine-needle aspiration (FNA) or biopsy is useful for histological diagnosis before surgery. However, the diagnostic yield and sensitivity of EUS-guided FNA are low, and its accuracy is reliant on the skill of the endoscopist [8,9]. In addition, this technique can cause serious complications such as intramural bleeding, perforation, or peritoneal dissemination of tumors. In this case, EUS showed typical findings of GIST, and endoscopy showed a central depression; therefore, bite-on-bite biopsy was performed due to suspicion of GIST. Although a small amount of mucosal bleeding occurred, the examination was terminated after hemostasis was confirmed, and the pathological results confirmed a GIST.

Due to the unique anatomical structure and characteristics of the duodenum, surgical treatments for duodenal GISTs may vary depending on its location, size, and involvement of adjacent organs, provided there is no distant metastasis [10]. Surgical treatment for duodenal GIST can be divided into limited and extended resection [11,12]. Limited resection includes local resection or wedge resection and segmental resection, while extended resection includes Whipple's or pyloric preserving operation after pancreaticoduodenectomy(PD). The surgical principle for GIST is complete resection of negative margins. Reports indicate that PD is occasionally performed instead of limited resection (LR), which is associated with a higher rate of surgical and postoperative complications, increasing morbidity and mortality. It has also been reported that extended surgery is not superior in terms of oncology [13]. Descending duodenal GIST has a high likelihood of requiring PD. This is because of the risk of periampullary organ injury, or when PD seems to occur when the duodenal GIST is large, involves the ampulla, or is likely to damage the PBD during minimal resection. If limited resection of the descending duodenal GIST can be performed without PD surgery, various complications can be prevented. There was no significant difference in prognosis compared to PD; therefore, LR was considered equally effective [14].

Minimally invasive surgery is difficult to perform for duodenal GISTs because of their anatomical location and difficulty in anastomosis after resection. As this tumor was located in the descending duodenum, there was a possibility of damage to the periampullary organs during laparoscopic surgery. Therefore, ENBD was placed before surgery to prevent damage to the periampullary organs, allowing real-time verification of the ENBD catheter during laparoscopic wedge resection. After surgery, ENBD tubography was performed to confirm the integrity of the anastomosis and check for periampullary organ injury. By performing laparoscopic wedge resection after inserting the ENBD catheter, the risk of PBD injury during surgery was actually reduced. SET near the AoV gives the advantage of preoperative ENBD insertion as above, but may be limited in some cases. For example, in cases where ENBD procedure is difficult, such as in cases of PBD benign stricture or large periampullary diverticulum, or in cases where complications due to ENBD procedure are high due to advanced age or serious underlying diseases, this procedure may be risky. Decisions need to be made considering risk and benefit.

Although this approach cannot be applied to all descending duodenal GISTs, inserting the ENDB before surgery can allow the extended operation to be converted to a limited one and open operation to a minimally invasive one.

## 4 Conclusion

To the best of our knowledge, this is a rare case of a patient with descending duodenal GIST treated with laparoscopic wedge resection after preoperational ENBD insertion. Although various situations should be considered, we believe that this approach will facilitate limited and minimally invasive surgeries for the treatment of descending duodenal GISTs, as demonstrated in this case.

## CRediT authorship contribution statement

Lee WY were attending doctors for the patient. Lee WY performed the surgical operation and endoscopist prepared the endoscopic report. Lee WY organized the report and wrote the paper. The author was involved in drafting and revising the manuscript, and author read and approved the final manuscript.

## Consent

Written informed consent was obtained from the patient for publication of this case report and accompanying images. A copy of the written consent is available for review by the Editor-in-Chief of this journal on request.

## Ethical approval

This is a case report; therefore it did not require ethical approval from ethics committee.

## Guarantor

Lee Woo Yong

## Provenance and peer review

Not commissioned, externally peer-reviewed.

## Sources of funding

This study did not receive any funding support.

## Registration of research studies

Not applicable.

## Declaration of competing interest

The authors declare no conflict of interest.
